# Intermediate Syndrome Presenting as Extrapyramidal Oromandibular Dystonia Due to Organophosphorus Inhalation: A Rare Case Report

**DOI:** 10.7759/cureus.51137

**Published:** 2023-12-26

**Authors:** Sarang S Raut, Saket Toshniwal, Vinit Deolikar, Sunil Kumar, Sourya Acharya

**Affiliations:** 1 General Medicine, Jawaharlal Nehru Medical College, Datta Meghe Institute of Higher Education and Research, Wardha, IND

**Keywords:** type ii syndrome, oromandibular dystonia, extrapyramidal, intermediate syndrome, organophosphorus

## Abstract

A 35-year-old male patient, a farmer by occupation, presented with a complaint of deviation of the angle of the mouth towards the right side while speaking. A cerebrovascular event was suspected in this patient. He had a history of exposure to propane and cypermethrin while spraying insecticide in his field. He has a history of chronic exposure to these compounds. Intermediate syndrome (IMS) also known as type II syndrome was diagnosed in this patient with the help of neuroimaging. On evaluation, the patient was found to be having oromandibular dystonia, which is an extrapyramidal symptom of type II syndrome.

## Introduction

Organophosphate exposure can develop complications such as acute cholinergic crisis, intermediate syndrome (IMS, also called type II syndrome), and organophosphorus-induced delayed polyneuropathy [1]. Three types of neuromuscular weakness can result from acute organophosphorus poisoning (OPP): type I syndrome, which is characterized by cholinergic signs; type II syndrome, also known as IMS, which is a delayed muscle weakness characterized by cranial, proximal limb, trunk, and respiratory muscle weakness; and type III syndrome, which includes polyneuropathy that develops one to four weeks after consuming particular organophosphates [2]. Type II syndrome is a "spectrum" of disorder in which the neuromuscular junction (NMJ) malfunction is brought on by OPP and develops gradually over time through a series of electrophysiological alterations that occasionally resolve quickly and commonly present as respiratory distress, leading to respiratory failure, and may require ventilation [3]. Characteristic features of type II syndrome are respiratory muscle weakness: diaphragm, intercostal muscles, and accessory muscles, including neck muscles and proximal limb muscles, with weakness of muscles supplied by some cranial nerves. It manifests two to four days after exposure when symptoms of acute cholinergic crisis have disappeared [4].

Extrapyramidal neurological symptoms are rare symptoms due to the involvement of basal ganglia bilaterally in IMS or type II syndrome [5]. The early detection of neurological complications such as extrapyramidal symptoms due to basal ganglia involvement in OP is facilitated by novel signal abnormalities on MRI [6]. This syndrome may worsen, resulting in pulmonary edema, respiratory failure requiring ventilator assistance, and respiratory tract infections. Additionally, there are cases reported in which IMS can be complicated, such as platypnea-orthodeoxia syndrome, a possible complication of IMS [7]. In patients with OPP and IMS who are on mechanical ventilation and are difficult to wean off, especially in patients with prolonged alcohol consumption, Marchiafava-Bignami disease characterized by necrosis and demyelination is also reported [8]. Inhaled organophosphate has also presented as superior laryngeal nerve palsy, which was improved after treatment with atropin [9]. Acute OPP has also presented with acute hemiplegia because of the overactivity of cholinergic and nicotinic receptors, causing increased sympathetic or parasympathetic activity [10]. Excessive cholinergic stimulation in the pancreas in OPP can present with acute pancreatitis and serum amylase levels can predict outcomes in OPP cases as serum amylase levels show a significant correlation with clinical outcomes in OPP cases [11].

## Case presentation

A 35-year-old male patient, a farmer by occupation, presented with complaints of tremors in both upper and lower limbs and deviation of jaw towards the right side while speaking for one day. This patient had a history of chronic exposure to propane and cypermethrin (OP compounds used as insecticides in fields) because he regularly sprayed these compounds in his fields. The patient was non-alcoholic and had no other comorbidities. On general examination, the patient was lying supine, afebrile to touch, and his pulse rate was 68 beats per minute, and oxygen saturation was 98 % on room air. There was no pallor, no icterus, no clubbing, no cyanosis, no lymphadenopathy, and no pedal edema. The pupils of the patient were 3 mm reactive to light, and fasciculations were absent. On cardiovascular examination, first and second heart sounds were heard with no murmur. Respiratory system examination was also normal with bilateral air entry equal, with no adventitious sounds. Per abdomen was soft and non-tender. Following a CNS examination, the patient was conscious and was oriented to time, place, and person; tone increased in all four limbs, power was grade 5 in all four limbs, deep tendon reflexes were present, and superficial tendon reflexes were present, with bilateral flexor plantar. All the cranial nerves were thoroughly examined, and it was normal. There is no deviation of mouth when the patient is not speaking, no ptosis, and no loss of taste sensation, which was suggestive of normal facial nerve function. Deviation of mouth was present while speaking. His initial laboratory investigation has been highlighted in Table 1.

**Table 1:** Laboratory investigations of the patient

Lab parameters	Observed values	Normal range
Haemoglobin	10 g%	13-17 g%
Total leucocytes count	10,000 cell/mm^3^	4000-11,000 cell/mm^3^
Platelet counts	3.19 cell/mm^3^	150,000-400,000 cell/mm^3^
Mean corpuscular volume	22.7 fL	83-101 fL
Creatinine	2.7 mg/dL	0.66-1.25 mg/dL
Urea	15 mg/dL	19-43 mg/dL
Sodium	139 mmol/L	137-145 mmol/L
Potassium	3.8 mmol/L	3.5-5.1 mmol/L
Alkaline phosphatase	95 U/L	38-126 U/L
Aspartate transaminase	43 U/L	17-59 U/L
Alanine transaminase	24 U/L	<50 U/L
Albumin	4.1 g/dL	3.5-5 g/dL
Total bilirubin	0.5 mg/dL	0.2-1.3 mg/dL
Serum acetyl-cholinesterase	0.2 U/mL	5.90-12.2 U/mL

**Figure 1:**
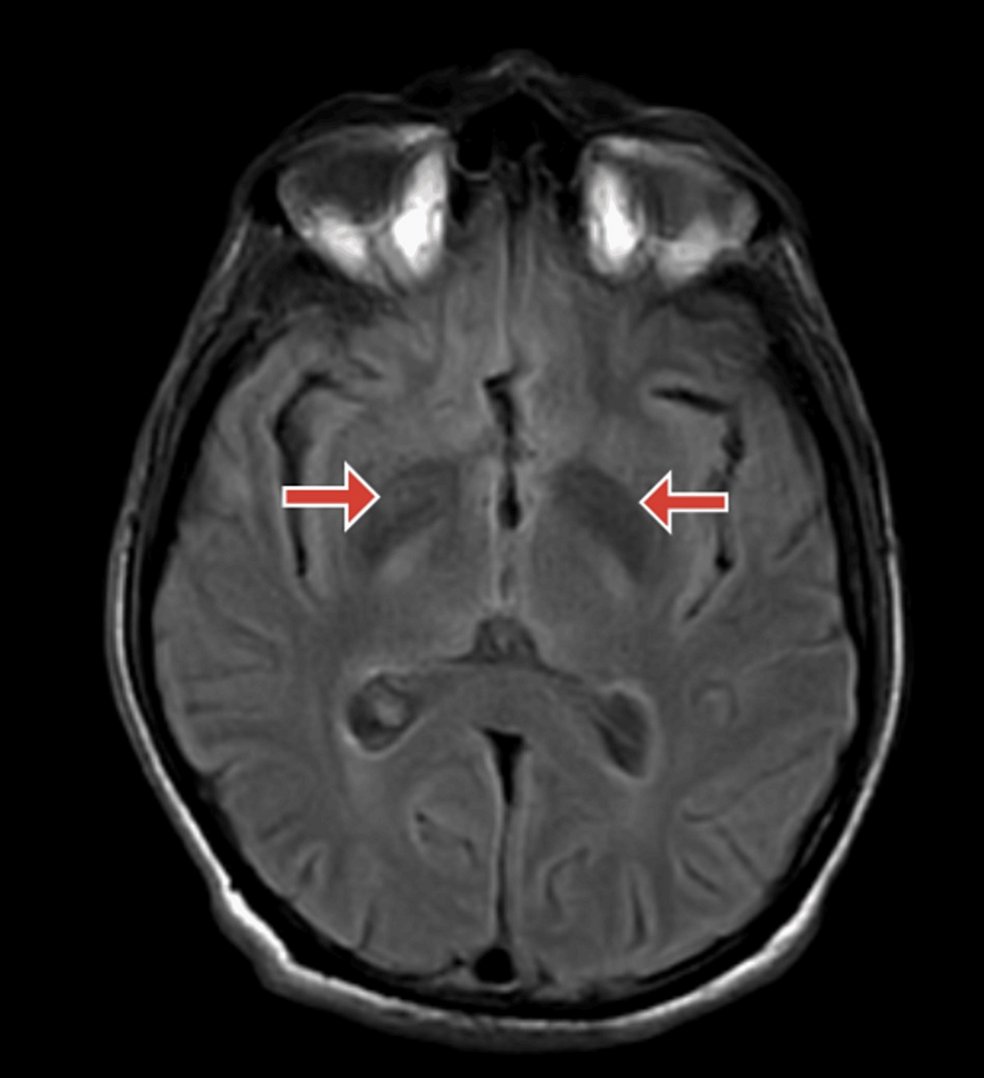
Magnetic resonance imaging (MRI) fluid-attenuated inversion recovery (FLAIR) section of the brain MRI brain FLAIR section showing symmetrical restricted diffusion in basal ganglia (red arrows).

**Figure 2:**
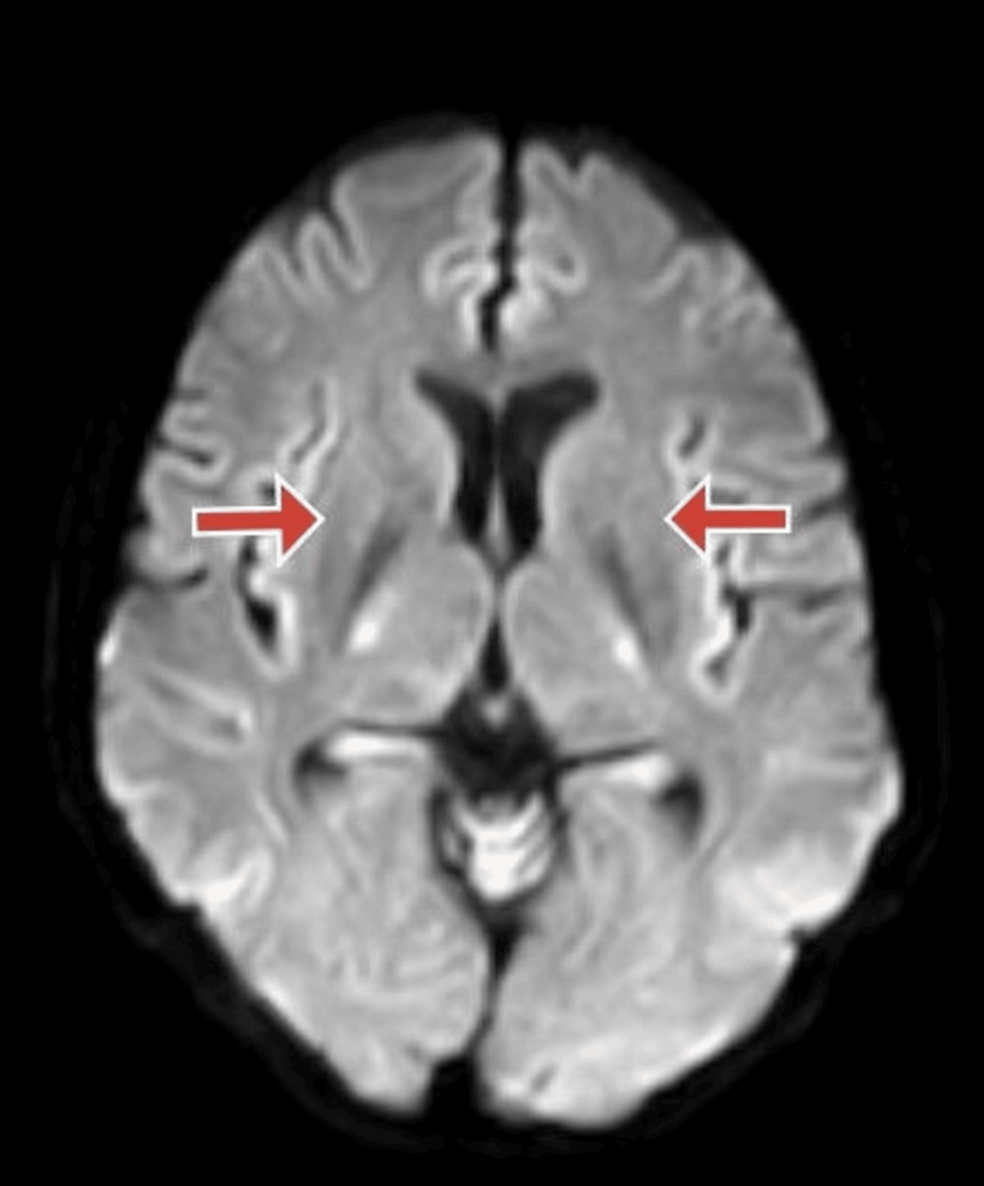
Diffusion-weighted magnetic resonance imaging (DW-MRI) section of the brain DW-MRI section of brain showing restricted diffusion in bilateral basal ganglia.

**Figure 3:**
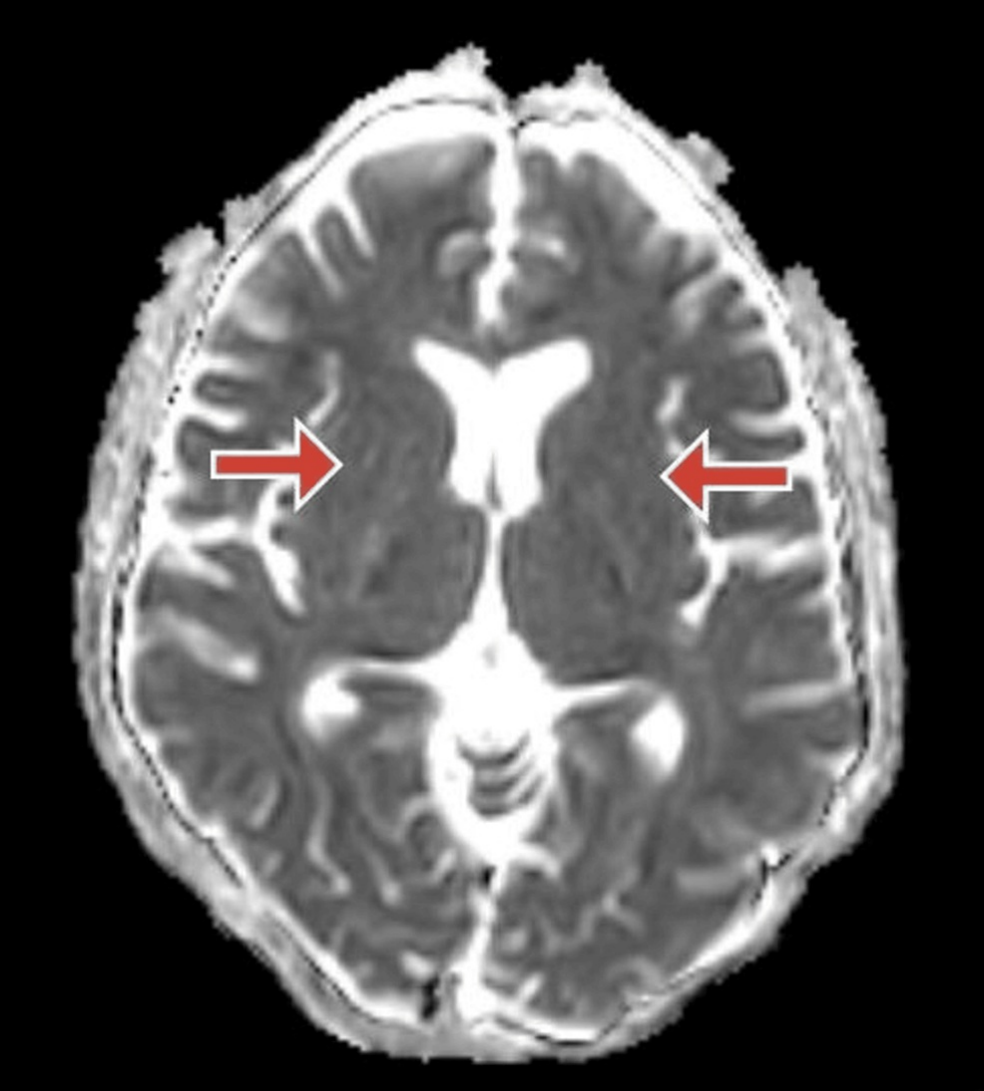
Magnetic resonance imaging apparent diffusion coefficient (MRI-ADC) section of the brain MRI-ADC section of the brain showing restricted diffusion in bilateral basal ganglia.

Electrocardiography of the patient was done suggestive of normal sinus rhythm. Magnetic resonance imaging (MRI) brain of the patient was done, which was suggestive of near symmetrical restricted diffusion in basal ganglia suggestive of IMS, as shown in Figures 1-3.

The patient was started with an injection of pralidoxime 2 g intravenous (IV) in 0.9% normal saline over 30 minutes, followed by a 1 g IV infusion in 0.9% normal saline for 48 hours. The patient was given injections of atropine 2 mg, 4 mg, 8 mg, and 16 mg IV bolus every five minutes for a total of 30 mg. Then, he was started with injectable atropine IV infusion at the rate of 3 mg per hour, injection of lorazepam 2 mg IV given every six hours for sedation, injection of pantoprazole 40 mg IV given once a day, and injection of ondansetron 4 mg IV given every eight hours. Symptoms of the patient subsided in 48 hours.

## Discussion

IMS, a neurological complication of OPP, occurs after resolving signs and symptoms of acute cholinergic crisis and before the onset of organophosphorus-induced delayed polyneuropathy, which is a late complication of OPP. Neuroimaging can be remarkable and can be apparent during IMS. Extrapyramidal symptoms, which occur infrequently as part of the IMS, are thought to be caused by acetylcholinesterase inhibition in the human extrapyramidal areas. In the absence of efficient detoxification pathways, increased susceptibility of the basal ganglia nuclei to toxic products may also be to blame. This condition cannot be treated with atropine or pralidoxime. However, abnormal signal intensities in the basal ganglia region have been seen in some patients' brain MRIs [12].

In this case, the patient presented with a history of exposure to organophosphorous compounds because of spraying insecticides in his fields, and he complained of tremors in both upper and lower limbs and deviation of jaw towards the right side while speaking. The patient did not have any signs of acute cholinergic crisis and no clinical signs of respiratory muscle weakness. However, he was having tremors in all four limbs, and oromandibular dystonia (the patient was having a deviation of jaw towards the right side while speaking) which are extrapyramidal symptoms. The serum acetylcholinesterase levels of the patient were deficient. We suspected a cerebrovascular event in this patient, and an MRI of the brain of the patient was done, which was suggestive of near symmetrical restricted diffusion in basal ganglia suggestive of type II syndrome, which is a well-known sequelae of OPP. Here, neuroimaging helped in the diagnosis of the type II syndrome with which the patient presented [5].

## Conclusions

Patients presenting with a history of exposure to OP compounds and presenting without any signs and symptoms of acute cholinergic crisis and having extrapyramidal symptoms should be considered for diagnosing type II syndrome, which is IMS. Brain imaging such as an MRI brain can help in diagnosing the IMS and should be considered in IMS.
